# Personalized Medicine in Asthma: Current Approach and Future Perspectives

**DOI:** 10.3390/jpm13101459

**Published:** 2023-09-30

**Authors:** Santi Nolasco, Claudia Crimi, Raffaele Campisi

**Affiliations:** 1 Respiratory Medicine Unit, Policlinico “G. Rodolico-San Marco” University Hospital, 95123 Catania, Italy; nolascos@hotmail.it (S.N.); raffaelemd@hotmail.it (R.C.); 2 Department of Clinical and Experimental Medicine, University of Catania, 95123 Catania, Italy

Asthma is one of the most common chronic respiratory diseases, affecting over 300 million people worldwide [1]. It is defined by intermittent bronchospasms that cause symptoms such as wheezing and dyspnea, and is characterized by airway inflammation, hyperresponsiveness and mucus hypersecretion, all contributing to often variable airflow obstruction [1]. The combination of these features can vary among patients, leading to differences in clinical presentation, known as *phenotypes*. Furthermore, the increased understanding of the immuno-pathophysiology of asthma recently allowed us to identify multiple disease *endotypes* through clinical biomarkers like sputum and blood eosinophils, serum IgE and fractional exhaled nitric oxide (FeNO) [2,3].

In the past, asthma was treated using a “one-size-fits-all” approach, where patients received the same medications regardless of their specific characteristics, and treatment primarily addressed symptoms without targeting the underlying inflammatory processes (Figure 1). However, the introduction of biomarkers has advanced the concept of precision medicine, which now takes into account the individual *pheno-endotype* of each patient [4,5].

The greatest contribution to personalized asthma care came from research on monoclonal antibodies (mAbs), which are used to treat the more severe forms of the disease, especially those with type 2-high inflammation (Figure 1). In this regard, omalizumab (anti-IgE) was approved for allergic asthma patients with high IgE serum values [6]; mepolizumab, reslizumab and benralizumab (anti-IL-5/Rα) are used for patients with severe eosinophilic asthma [7]; and dupilumab (anti-IL-4Rα) is used for patients with high eosinophil and/or FeNO values [8]. Clinical trials have shown that these drugs not only reduce the number of exacerbations, but also limit the noxious exposure to long-term oral corticosteroids, stabilize respiratory function and improve overall asthma control [9]. Real-world studies have also significantly increased the understanding of the optimal patient for each severe asthma monoclonal antibody, allowing for subjects with more heterogeneous characteristics to be compared. The most relevant example is chronic rhinosinusitis with nasal polyposis, a comorbidity that affects up to 60% of people with severe eosinophilic asthma. In real-world studies, it has been shown that the presence of nasal polyps improves the response to asthma outcomes in the case of anti-IL-5/Rα and anti-IL-4Rα therapy [10,11]. Another interesting scenario is the co-presence of asthma and bronchiectasis, a chronic disease characterized by a permanent enlargement of the airways [12,13]. Bronchiectasis affects nearly 30–50% of patients with severe asthma, compared to 3–10% of milder forms of asthma, and is associated with more exacerbations, worse respiratory function and higher oral corticosteroid use [14]. Since eosinophils and type 2 inflammation contribute to the processes of airway damage and remodeling, recent studies have shown that mepolizumab or benralizumab treatment can potentially limit the mixed neutrophilic–eosinophilic inflammation and block the vicious cycle of airway damage and remodeling [15,16,17]. 

However, to date, only severe asthma patients receiving mAbs are treated with the most tailored approach, since biomarker measurement and comorbidity assessment have become essential for prescription and response prediction. Lately, researchers have proposed the idea of using severe asthma mAbs in some patients with mild type 2-high asthma [18] with the aim of preventing airway remodeling and disease progression [19]. This novel approach might facilitate the achievement of asthma *remission*, an ambitious goal that is still debated among academics about its definition and applicability, which are based on a set of composite outcomes, including no exacerbations, good disease control, no oral corticosteroid consumption and stabilization of respiratory function [20,21,22]. Nonetheless, it is also important to consider that the high cost of biological drugs makes it very complicated to expand the number of potential recipients.

Still, much remains to be done, as the currently validated biomarkers only predict responses in a subset of patients. Recent studies showed that high values of IL-5, eosinophilic peroxidase and thymic stromal lymphopoietin (TSLP) in sputum, which indicate “hot” airways, predicted clinical remission at 12 months in a larger percentage of cases compared to classical biomarkers such as the eosinophil count in peripheral blood, serum IgE and FeNO [23]. These data encourage clinicians to become better prescribers by enhancing their predictive skills, thus improving their understanding of the inflammatory processes that govern this disease at any stage of severity.

In this regard, airway imaging has the potential to further push the envelope of precision medicine in asthma treatment. In 2018, Dunican and colleagues showed the impact of mucus plugs on asthma severity and highlighted the central roles of eosinophil peroxidase and mucus hypersecretion as key pathogenic features [24]. These findings have since served as catalysts for recent studies that revealed that both benralizumab and dupilumab effectively reduced mucus plugs and improved lung ventilation as measured via ^129^Xe MRI [25]. Thus, using these drugs may be considered a smart choice to treat type 2-high severe asthma with concomitant mucus plugs [26,27,28].

Functional, clinical and imaging biomarkers are also crucial in the diagnosis and treatment of asthma–COPD overlap. In this Special Issue, Alsayed et al. discussed the recent evidence on this topic, highlighting the potential role of mAbs, which are currently used as a viable treatment option for severe asthma [29], as supported by the positive results of the phase 3 BOREAS trial on dupilumab for COPD patients with eosinophil counts of ≥300 cells/µL [30]. These recent findings expand the horizons of precision medicine beyond the treatment of a single disease to target underlying inflammatory processes [31].

In conclusion, despite there being many unmet needs, the future looks promising for both severe asthma patients and physicians. The latter will have access to an increasing number of tools to understand and treat this condition, while asthma patients will benefit from more effective therapies aimed at completely changing the natural history of this disease. In this regard, tezepelumab, a novel mAb that blocks the upstream TSLP cytokine, was recently approved by the FDA for the treatment of severe asthma without any biomarker limitations [32,33], providing a treatment option for patients with type 2-low asthma for the first time [34,35]. Nonetheless, the good practice of assessing as many biomarkers as possible in each patient to determine the correct *pheno-endotype* should be supported and encouraged in the process of creating personalized treatment.

## Figures and Tables

**Figure 1:**
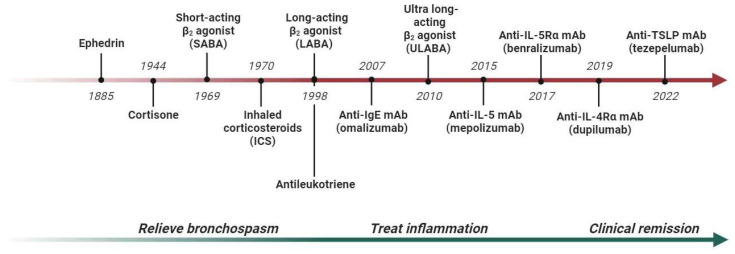
Development of asthma treatment and outcomes from the 19th century to the 21st century. Abbreviations: IL, interleukin; mAb, monoclonal antibody.
